# Organoid assessment technologies

**DOI:** 10.1002/ctm2.1499

**Published:** 2023-12-19

**Authors:** Yuyuan Gu, Wencai Zhang, Xianmin Wu, Yuanwei Zhang, Ke Xu, Jiacan Su

**Affiliations:** ^1^ Institute of Translational Medicine Shanghai University Shanghai China; ^2^ Organoid Research Center Shanghai University Shanghai China; ^3^ School of Medicine Shanghai University Shanghai China; ^4^ Department of Orthopedics First Affiliated Hospital Jinan University Guangzhou China; ^5^ Department of Orthopedics Shanghai Zhongye Hospital Shanghai China; ^6^ Department of Orthopaedics Xinhua Hospital Affiliated to Shanghai JiaoTong University School of Medicine Shanghai China; ^7^ Wenzhou Institute of Shanghai University Wenzhou China

## Abstract

Despite enormous advances in the generation of organoids, robust and stable protocols of organoids are still a major challenge to researchers. Research for assessing structures of organoids and the evaluations of their functions on in vitro or in vivo is often limited by precision strategies. A growing interest in assessing organoids has arisen, aimed at standardizing the process of obtaining organoids to accurately resemble human‐derived tissue. The complex microenvironment of organoids, intricate cellular crosstalk, organ‐specific architectures and further complicate functions urgently quest for high‐through schemes. By utilizing multi‐omics analysis and single‐cell analysis, cell‐cell interaction mechanisms can be deciphered, and their structures can be investigated in a detailed view by histological analysis. In this review, we will conclude the novel approaches to study the molecular mechanism and cell heterogeneity of organoids and discuss the histological and morphological similarity of organoids in comparison to the human body. Future perspectives on functional analysis will be developed and the organoids will become mature models.

## Graphical abstract

**•** Technologies to assess the stability and authenticity of organoids

**•** From the mechanism to the functional evaluation of organoids, compared to human organ/tissue in vivo

**•** Single‐cell technologies and functional property technologies are important to assess the organoids both at macro‐ and micro‐level

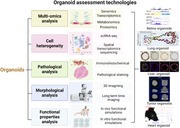

## 1 INTRODUCTION

The term ‘organoid’ has been used to define complex three‐dimensional (3D) structures, which displays similar architectures and functionalities to in vivo organs.^1^ Now, a more restricted definition encompasses organoids generated from primary tissues or stem cells, serving as in vitro platforms to mimic multiple aspects of their organ counterparts beyond just their structures and functions. Advances in stem cell culture and understanding of extracellular matrix (ECM) biology have enabled the cultivation of organoids, capturing key multicellular and functional hallmarks of real organs. Prior to organoid technology, inducing stem cells to emulate real organs in 3D was limited due to immunogenic risks and functional integration challenges. However, the 3D organoid technique pioneers a new era that empowers researchers to study the molecular mechanisms of diseases and develop novel treatment options.

Organoids hold tremendous potential for biomedical applications including providing recapitulate models of biological development and various pathologies and predicting drug responses in a personalized fashion.^2^, ^3^ As a result, difficult interventional studies are given chance to conduct in human subjects. For instance, a study created primary and metastatic breast cancer (BC) organoid lines provided a valuable in vitro platform for BC research and drug discovery.^4^ Needless to say, organoids provide patient‐specific avatars including treatments for cancer, rare genetic diseases, and multifactorial disorders whereas the applications of organoids are only beginning to be explored.

Recent research has seen significant advancements in organoid construction for modeling diseases and human tissues, making it a burgeoning field.^2^, ^5^ Nevertheless, in order to exert full potential of organoid technologies, most immediately, better characterization and validation of organoids are still supposed to continually improve. Although local patterning in organoid models shows promise in producing relative positioning regions, macro‐scale arrangement still differs from real organs. Indeed, a very strong ‘batch effect’ that huge variability between terms of formation occurred when generating regional identities, although organoids made at the same time were highly similar, finally resulting in unstable quality.^6^ This will require multi‐faceted assessments for organoid models to set quality standards in cell composition and consistency in histological, morphological and functional responses to stimuli.

The structures of organoids are often heterogeneous and irreproducible, for example, missing cell types or aberrant gene regulation, limiting their use in the clinic. Multi‐omics analysis plays a pivotal role in fully assessing organoid models, revealing their molecular mechanisms at the genome, transcriptome, and proteome levels. Furthermore, single‐cell analysis and spatial profiling yield an unprecedented quantitative, high‐dimensional assessment of comprehensive molecular maps,^7^ providing reference atlases for disease‐centric studies. Pathological and morphological analysis restore the full picture of architectures on a macro‐scale to assess the similarity to in vivo organs and disease/drug models in vitro. These methods are wildly used and appropriately assess organoids from cellular level to structural level. Then, functional properties analysis will complete the final piece of the puzzle of whether organoid protocols could achieve the simulacrum with complex functions similar to real organ counterparts. In fact, it is particularly relevant for aggressive malignancies to screen drugs suited to the patient's genetic profile.^8^ Therefore, a future catalog of well‐assessed organoids would pay more attention to quantify biological variation in presenting primary tissue in vivo and further solving the environmental perturbations on organoids in vitro models.

Assessments of organoid models have significantly advanced our understanding, from mechanistic investigations to functional properties.^9^ Here, we reviewed recent applications of various analytic approaches to study drug responses and disease pathogenesis using organoids. We further discuss functional properties of organoids to assess their reliability as translational bridges between patients and animal research models, facilitating the translation of in vitro discoveries into clinical applications. Organoids hold promise in personalized and regenerative medicine, as they can produce functional biological structures for safe transplantation into patients.

### 1.1 Multi‐omics analysis

**FIGURE 1:**
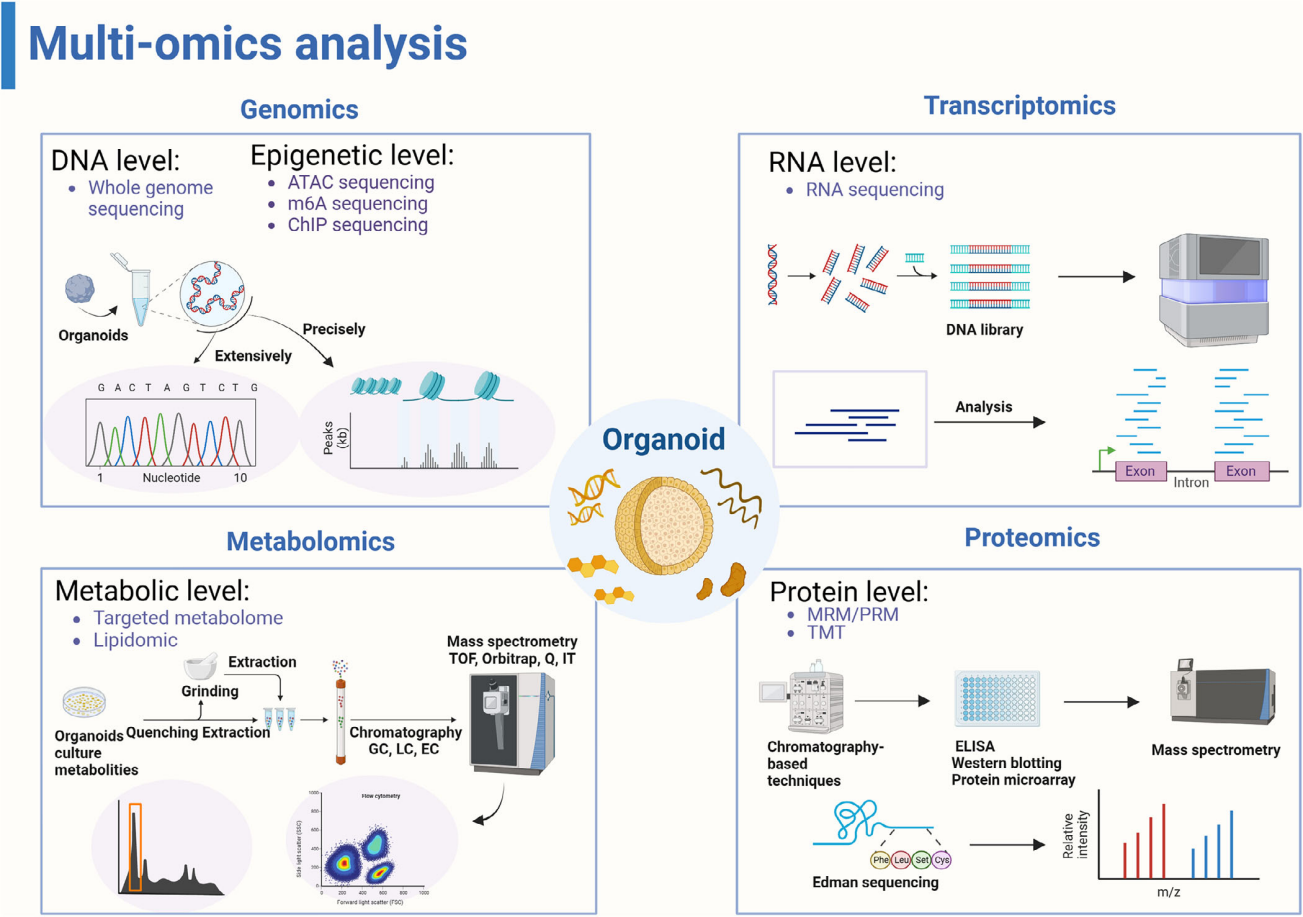
The applications of multi‐omics analysis in assessing organoids. Whole genome sequencing is widely used at the DNA and epigenetic levels to analyze gene maps of organoids, comparing their similarities. ATAC‐seq is frequently employed to discover precise epigenetic regulatory networks. RNA‐seq is a common method for evaluating molecular mechanisms. Additionally, proteomics and metabolomics analyze metabolites and co‐cultures of organoids, such as intestinal organoids. (Created with BioRender.com).

Methodological advances in next‐generation sequencing enhanced transcriptomic and epigenomic sequencing sensitivity (Figure 1). Multi‐omics analysis could assess transcriptomic and epigenomic expression to monitor stably inheritance of organoid lineages and organoid derivative can be assessed by proteomics and metabolomics sequencing.

### 1.2 Genomics

Genomics usually studies the genomic expression of organisms from a holistic aspect, which allows to test whether the organoid is directed to develop. Genomic studies help reassess the suitability of complex organoid culturing conditions. An assay was originally developed for assessing the overall genomic drifting of the cellular composition in contemporary recipes for primary breast cancer organoid long‐term culturing by whole‐genome sequencing. The results suggested that most representative tissue origins are better restricted to primary culture.^10^ Genomic analysis optimized directional differentiation conditions for inner organoids, allowing stable imitation of hepatocyte‐specific gene expression profiles in alpha‐1 antitrypsin deficiency carriers.^11^ Similarly, across brain organoid culturing models, microglia play a conservative role, regulating key transcriptional programs and inducing changes in neural stem cells in response to local culturing environments.^12^

Genomic analysis reveals the anatomical origin of organoids from different sources and that the transcriptomic profile is highly comparable in the same donors. For example, bile cholangiocyte organoids (BCOs) derived from the gallbladder exhibited similarities to in vivo cholangiocytes. However, regional‐specific gene differences were observed even in BCOs obtained from different sources of bile, suggesting that culture conditions mainly influence the general transcriptomic profile rather than the organoid's origin.^13^ The similarity in genomic profiles opens new doors to assessing the organoids for disease modeling without need of invasively collected biopsies.

Epigenetic technology is an appropriate technology to assess the degree to which diseases correlate with acceleration of aging hallmarks. The epigenetic clock is particularly suitable for those diseases with a strong inflammatory component. Organoids maintain segmental DNA methylation patterns and age stages during cellular differentiation, making them reliable tools for investigating stem cell‐intrinsic aging in vitro.^14^

### 1.3 Transcriptomics

Traditional transcriptomics is also suitable for organoid research. Compared to genomics, it pays more attention to whether organoids have specific functions after maturation.

**FIGURE 2:**
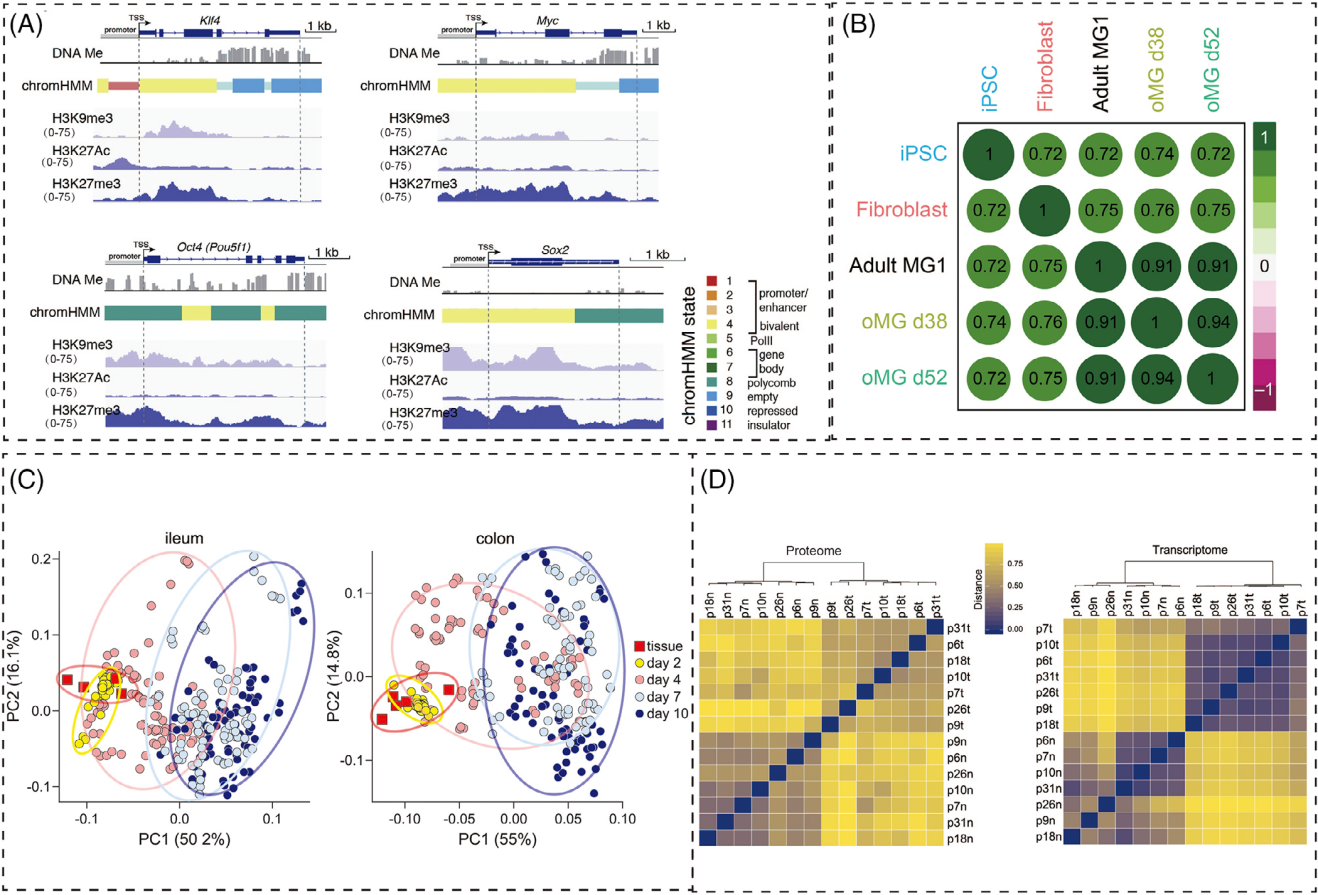
Multi‐omics analysis assesses organoids at molecular level. (A) Using DNA methylation, chromHMM and ChIP‐seq to trace the reprogramming genes expression level in retina organoids for H3K9me3, H3K27Ac, and H3K27me3.^32^ Copyright 2018, Elsevier. (B) The expression of characteristic cell surface markers of microglia is similar between organoid‐grown microglia (oMG) and adult MG.^18^ Copyright 2018, Springer Nature. (C) The differentiation of ASC organoids derived from the ileum and colon is similar and can be used as a baseline for gene expression in subsequent analysis.^19^ Copyright 2021, Elsevier. (D) Correlation heatmaps of the differentially expressed proteins from the proteomics (left) and transcriptomics (right) datasets in CRC Organoids.^20^ Copyright 2017, Elsevier.

High‐throughput organoid drug screening should ensure the concordance between the organoid models and their corresponding patient tumours. Using castration‐resistant neuroendocrine prostate cancer phenotype organoids, which were genomic and epigenomic concordance to parent patient tumours discovered that the enhancer of an epigenetic modifier, zeste 2 (EZH2). It involved in the pathogenesis of androgen‐independent mechanism.^15^ This nominated a drug combinations strategy in clinical trials for screening targeted drugs in rare cancer organoids models by manipulating the expression of oncogenes in the neuroendocrine phenotype.^16^ Additionally, genomic and phenotypic stability can also be systematically tested when gene modifications changed in organoid construction. LuCaP patient‐derived xenografts (PDX)/organoid models as a genotype‐dependent model to test drug sensitivity. They maintained the reliance on androgen receptor signaling pathway and genomic heterogeneity was also been conserved, which provided a genetically‐characterized platform to investigate disease pathogenesis as well as therapeutic responses.^17^ Ormel et al. validated the microglia identity of organoid‐grown microglia with a typical molecular phenotype, morphology and functions similar to adult microglia at the whole transcriptome level with RNA‐sequencing and flow cytometric analyses^18^ (Figure 2B). Thereby the more precise the whole gene network is, the better evaluation of the molecular mechanisms of organoid models to reveal the pathogenesis of the disease.

### 1.4 Metabolomics

The large variability in primary organoids cultures leads to increased noise in experimental systems because these cultures contain a variety of cell types, producing unexplained results. However, donors, differentiation medium, passages, and cell culture replication remain controllable. In intestinal biology, thoroughly evaluation of human organoid cultures requires meticulous quantification of various sources of variation, particularly metabolic activity in the gut. A study found that donor‐donor variability in adult human intestinal stem cell organoid cultures was maintained at manageable levels when evaluated by targeted analysis of central carbon metabolites and hormone production models, thereby enabling robust and interpretable experimental design for studying metabolic processes^19^ (Figure 2C).

### 1.5 Proteomics

Creating personalized human proteome profiles of organoids necessitates more sensitive and rapid proteomic evaluations. Expanding studies to include more patients and organoid proteome profiles will enhance disease classification and provide technical tools for optimizing personalized treatment. Compared with healthy organoid proteomes, expression levels of several proteins changed significantly and consistently in tumour organoids, which were previously reported as cancer biomarker proteins. Quantitative mass‐spectrometry‐based proteome profiles of patient‐derived tumour organoids recapitulated diversity among patients, which even resembled to original tumour.^20^ (Figure 2D)

Since phenotypic differences are closely related to changes in the proteome, personalized proteomes are supposed to be better evaluated. Proteomics analysis revealed diverse epidermal cell proliferation and differentiation proteins in iPSC‐derived epithelial and mesenchymal (EM) organoids. These proteins, present in skin tissues, contribute to various signaling pathways, supporting epidermal development. On the other hand, different ECM proteins in EM organoids could promote some cytokines and regulated factors for tissue repairing in the future.^21^ Discrepancy in individual tissue can also reflect in specific proteome. When exposed to hypoxia and reoxygenation (HR) organoids, intestinal lesions due to ischemia‐reperfusion (IR) in crypt‐like (CL) and villus‐like (VL) human intestinal organoids manifested differently. To decipher protein dynamics and the pathology of HR, Kip et al. used a system‐wide proteomics approach based on mass spectrometry (MS) and functional enrichment analysis. Significantly altering proteins showed that changes in protein metabolism were more pronounced in CL organoids while cell stress and cell death were more pronounced in VL organoids.^22^

Collectively, a deeper understanding of personalized patient‐specific organoid proteome profiles contributes to diagnosis of patients leading to the developments of personalized therapies.

### 1.6 Cell heterogeneity

**FIGURE 3:**
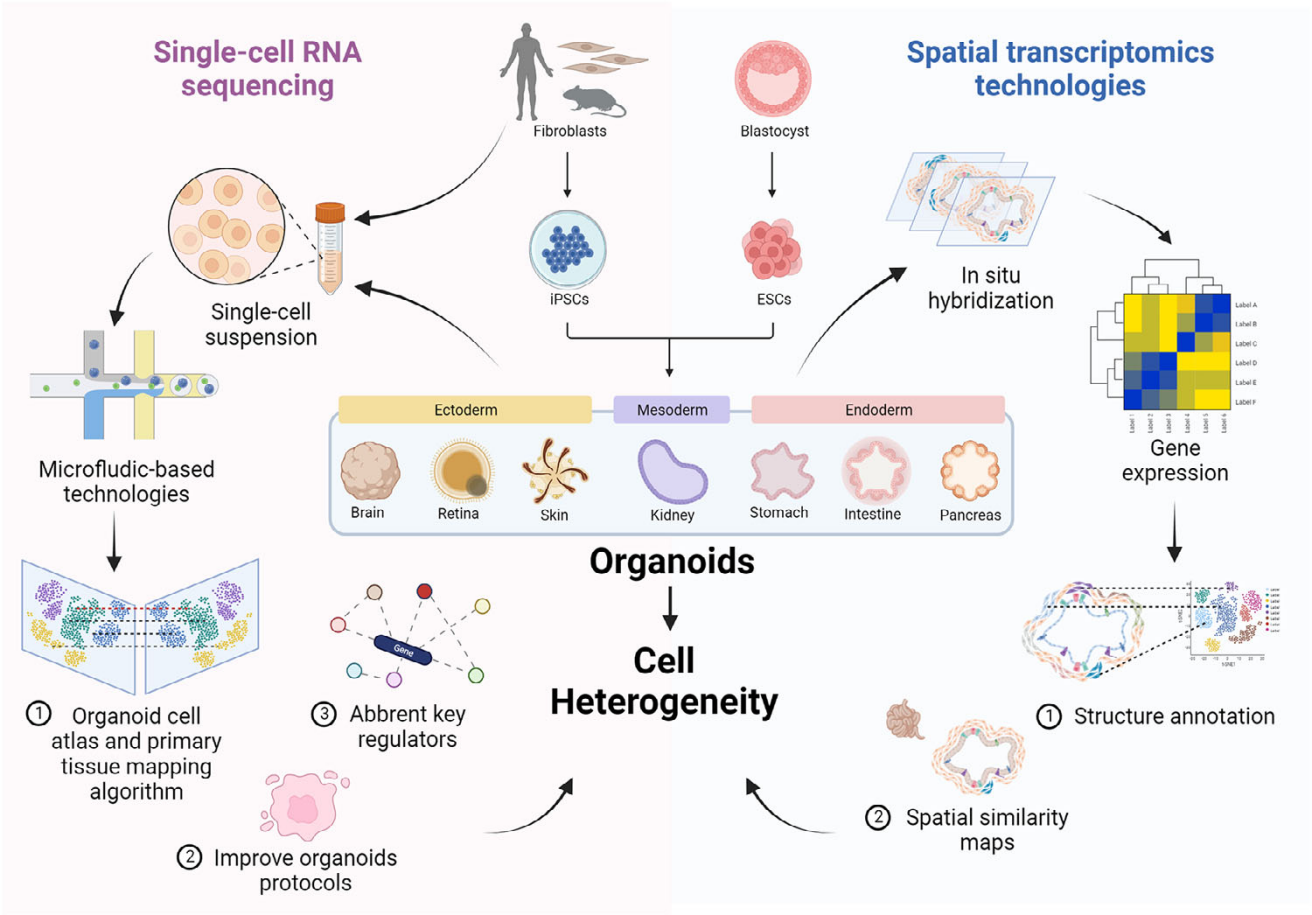
Single‐cell technologies decipher the cell heterogeneity of organoids. With the high‐throughput technical advances in single‐cell technologies, single‐cell RNA sequencing is widely used to decode the transcripts of the organoids and their counterpart organs to find the mutated key regulators or improve the constructing protocols. Spatial transcriptomics technologies are suitable for 3D multiple cellular structures to annotate spatial information. (Created with BioRender.com).

Model organisms possess physiologically complete systems, but they do not have human genetic background. Organoids are more suitable for disease presentations and high‐throughput screening.^23^ However, in complex and diverse heterocellular systems, organoids are particularly well suited for the use of emerging high‐dimensional technologies, such as single‐cell technologies and spatial profiling. These methods offer reproducible assessment techniques as predefined standards for assessing the organoid models enabling comprehensive deciphering of underpinning single‐cell and single‐organoid heterogeneity in a research laboratory setting (Figure 3). Furthermore, the fusion of single‐cell technology and organoid technology, two rapidly developing fields, is not far away.^24^

### 1.7 Single‐cell RNA sequencing

The diversity of organoid models is still a concern when used in disease modeling: how can researchers detect organoid genotypes associated with disease phenotypes? Thorough analysis is crucial to understand the mechanisms governing organoid formation and their relevance to in vivo development.

#### 1.7.1 Tumour organoids

**FIGURE 4:**
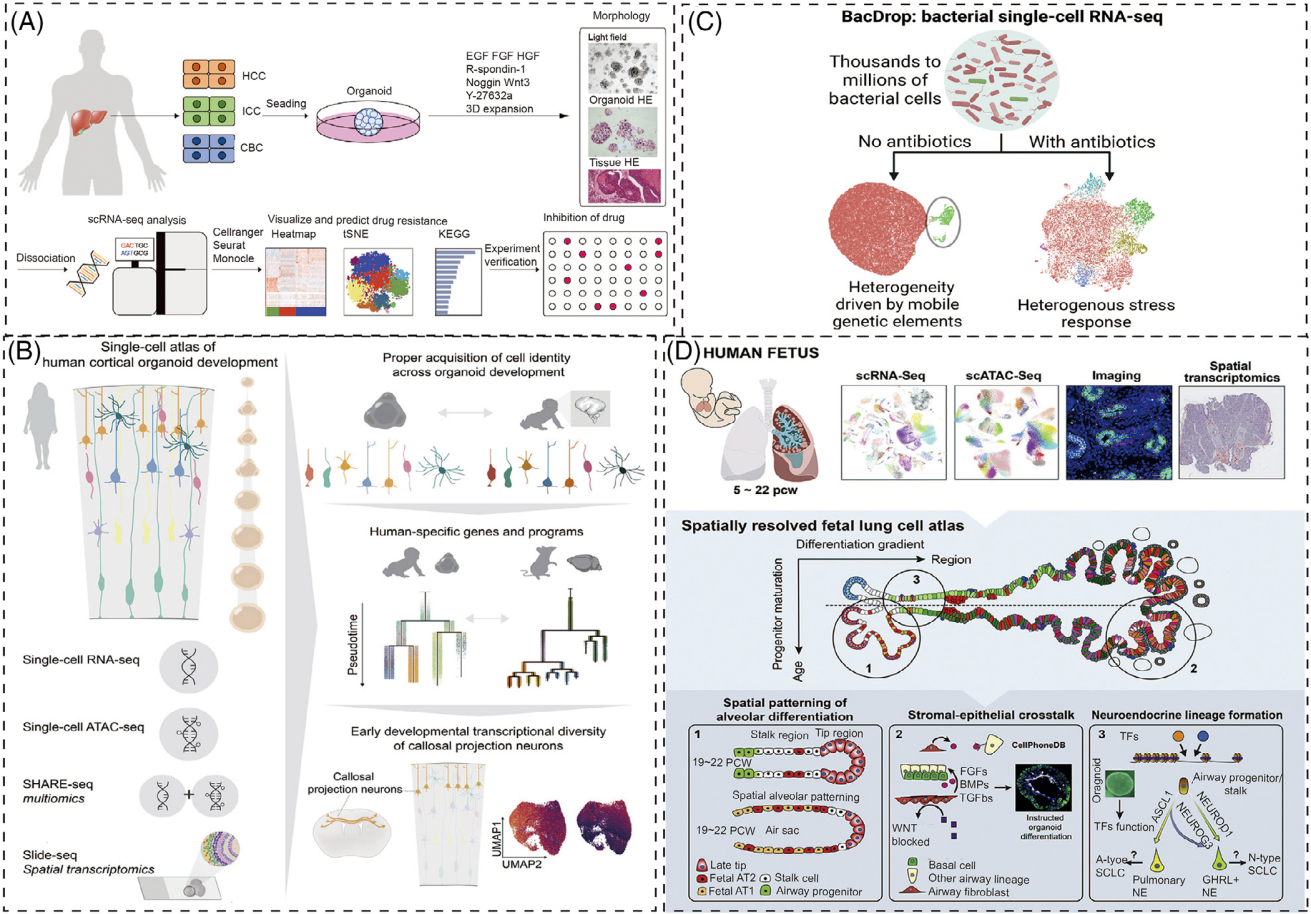
The applications of single‐cell technologies in different organoid models. (A) scRNA‐seq reveals intra‐tumoural heterogeneity and mechanisms for drug resistance in hepatobiliary tumour organoids.^26^ Copyright 2021, Wiley‐VCH GmbH. (B) The single‐cell atlas of human cortical organoid development is assessed by single‐cell transcriptomic, epigenetic, and spatial information.^30^ Copyright 2022, Elsevier. (C) Bacterial droplet‐based scRNA‐seq reveals heterogeneous cellular states.^38^ Copyright 2023, Elsevier. (D) Coupling single‐cell methods with spatial analysis discovered a multi‐omic cell atlas of human lung development tested by organoid models.^55^ Copyright 2022, Elsevier.

The heterogeneity in cell‐level behavior within tumour organoids is of clinical importance especially relevant to develop new therapeutics.^25^ Single‐cell analysis is firstly used in this field to assess the drug response in tumour organoid. Molecular heterogeneity of hepatobiliary tumour is important to discuss drug resistance including intertumoural and intra‐tumoural disparity. Zhao et al. used single‐cell RNA sequencing to explore heterogeneity and evolution of hepatobiliary tumour organoids responding to drug resistance. They proposed that the collaboration of intra‐tumoural heterogenic subpopulations rendered malignant phenotypes.^26^ (Figure 4A)

Single‐cell techniques also revealed baseline metabolic heterogeneity across cells within individual organoids in the same culture, independent of growth heterogeneity. Automating assessment methods with patient‐specific data from PDCOs can normalize baseline culture characteristics and enable robust measurements, testing clinically relevant drug doses and schedules for potential successful therapies.^3^ Close to the links of diversity of normal cell types, presence of multiple structure types with structural heterogeneity are notable in human organoid cultures. Single‐cell analyses united with mass cytometry (cytometry by time of flight [CyTOF]) extensively evaluated the ability of organoid culture whether retained complex progenitor or differentiated cell types via long‐term propagation. It has been demonstrated that the multiple mammary epithelial lineages in organoids faithfully preserved the mammary‐specific protein expression patterns of original tissues in a single culture.^27^

#### 1.7.2 Brain organoids

In order to simulate the complexity of brain, which is often composed of multiple subpopulations of cells. Single cell sequencing technology has become an indispensable evaluation tool for evaluating brain organoids to detect the genetic map of brain. The distinct characters of human cortical development, such as progenitor maturation trajectories and areal specification of newborn neurons, were not fully recapitulated in organoids according to scRNA‐seq data. Ectopic activation of cellular stress pathways impaired cell type specification, scRNA‐seq datasets offer an initial framework for evaluating the accuracy of cortical organoids as in vitro models of human brain development.^28^ However, the lack of specificity in organoids should be carefully considered when studying disease phenotypes with specific cell types. scRNA‐seq of pitrilysin metallopeptidase 1 (PITRM1)‐knockout iPSCs spontaneously developed brain organoids showed pathological features of Alzheimer's disease (AD). This examined the cell‐type‐specific pathogenetic pathogenesis of PITRM1 deficiency, supporting the mechanical association between mitochondrial function and neurodegenerative protein diseases.^29^ Uzquiano et al. present the comprehensive and precise cell atlas of human cortical organoid development from single‐cell transcriptomic, epigenetic and spatial information, which brings the human corticogenesis in vitro from generation of neural progenitors through production of differentiated neuronal and glial subtypes^30^ (Figure 4B).

#### 1.7.3 Retinal organoids

Unlike the real human retina, currently used retinal organoids unable to transmit light responses synaptically to inner retinal layers. Additionally, modeling genetic diseases of the retina lacks a quantitative comparison of gene expression. Retinal organoids urgently need high‐throughput sequencing technology to detect their genetic characteristics. scRNA‐seq of human embryonic stem cells (hESCs)‐derived retinal organoids from five differentiation time points showed a rich pedigree information of transcriptome. Wang et al. also identified insulin receptor, a specifically expressed receptor, has robust effects on the genesis of photoreceptors.^31^ Extending transcriptomic analyses with scATAC‐seq can help assess potential gene regulatory networks in the retina.^32^ However, accessible chromatin in hESC‐derived retinal organoids differs from human fetal development, lacking specializations like the fovea, and reveals differences in Notch signaling dysregulation. To closely resemble the human retina, precise regulation of relevant signaling pathways may improve the relative cell composition of retina organoids.^33^ Cis‐regulatory elements (CREs) was implicated in several inherited disorders in the retina. The combination of snATAC‐seq and snRNA‐seq has been effective in identifying developmental dynamics and cell‐class‐specific CREs in the human retina and iPSC‐derived retinal organoids. This suggests the significant potential of organoids as a valuable model to study CRE‐related diseases.^34^

To further assess the fidelity of retinal organoids, the resource of cell line to produce retinal organoids should consider their timing and efficiency of the organoid development. Protocols of rhesus macaque iPSCs (rhiPSC) to establish retinal differentiation organoids was demonstrated with scRNA‐seq. RhiPSC‐derived retinal organoids are characteristically indistinguishable from human iPSC‐derived retinal organoids, following a brief but similar, faster development.^35^

#### 1.7.4 Intestinal organoid

The originating cell states of intestinal organoid are inherently complex and poorly scaled, the applications to study complex molecular mechanisms of disease will be limited. Utilizing massively parallel scRNA‐seq, a generally applicable framework compares cell types and states in vivo with organoids, enhancing model fidelity.

Intestinal organoids are commonly used to study Paneth cell (PC) degranulation dynamics, but a global comparison of defined cell types and states is lacking. These organoids, formed by a single intestinal stem cell with self‐organizing ability, mimic diverse cell types and tissue organizations, creating complex multicellular asymmetric structures. Crucial in asymmetric structures conforming process is the first symmetry‐breaking event that a fraction of stem cells differentiate into PC. They used scRNA‐seq to discover unrecognized heterogeneity within PCs and demonstrated how the cell states adaptively changed responding to different infections.^36^

scRNA‐seq draws the picture of intrinsic self‐organized behavior of single cells when exposing to a uniform growth‐promoting environment. By leveraging the stress‐response factor Nupr1, which promotes the survival of PCs, the physiological fidelity of intestinal organoids improves. This enhancement is characterized by transcriptomic, cytometric, morphologic, and proteomic changes, leading to advanced mechanistic insights and bioengineering capabilities beyond in vivo tissue mapping.^37^ Recently, a highly scalable technology, BacDrop, for bacterial scRNA‐seq was developed for 5unlocking new microbiological insights into bacterial responses to intestinal organoids^38^ (Figure 4C).

#### 1.7.5 Kidney organoids

Hampered by variability, nephron immaturity, low throughput and limited scale, kidney organoids require for better methods to deliver rapid and high throughput generation with reproducible cell viability. A study applied extrusion‐based 3D cellular bioprinting in constructing kidney organoids and recognized cell clusters were assessed by gene/protein expression analyses of scRNA‐seq, showing similar in all organoid conformations. Additionally, greater total nephrons are formed in bioprinted lines and precisely manipulated with biophysical properties.^39^ Hence, automated extrusion‐based bioprinting methods delivered improvements in quality control and scale for kidney organoid production and scRNA‐seq provided a useful technology to assess the suitability of the construing methods.

Single‐cell technologies are playing a crucial role in advancing organoid differentiation protocols and distinguishing differences in varied protocols. The induction of SIX2+ nephron progenitor cells is crucial to generate functional nephrons for kidney organoids.^40^ Wu et al. compared two directed differentiation methods using scRNA‐seq and snRNA‐seq, analyzing transcriptomics from 65 organoids of fetal and adult kidney cells. By reducing neurons by 90%, they achieved diverse renal cell types without compromising organoid differentiation.^41^ They also mentioned that two different protocols, which generates SIX2+ (Sine oculis homeobox 2) nephron progenitors^42^ or induce PAX2+ (paired box 2) anterior intermediate mesoderm cells,^40^ would largely affect the complexity and maturation of kidney organoids. A recent study emphasized the importance of metanephric specification to generate more matured nephrons^43^ and enhanced kidney organoids with superior proximal tubules, which consistent with developmental studies that showed the distinct progenitor population for metanephros, functional kidneys.^44^ For disease study, snRNA‐seq was also used to elucidate intrinsic cellular repair mechanisms in kidney organoids.^45^

#### 1.7.6 Bone organoids

In 2022, the bone organoid‐related research was reported for the first time internationally and began to discover their potential use.^5^ Bone organoids refer to the use of osteoid matrix bioactive materials,^46^ through in vitro 3D culture combined with directional induction technology,^47^, ^48^ cultivate and assemble various stem cells (such as bone stem cells etc.) or functional cells (osteoblasts, osteoclasts, etc.) into osteoid tissues. Bone organoids have some specific functions of bone, which can be used for bone biological mechanism research,^49^, ^50^ drug screening and bone tissue regeneration and repair.^51^, ^52^ From the perspective of differentiation regulation, bone formation has a long‐range nature, and the cells are not exactly the same in different time profiles.

Bone organoids, unlike other types of organoids, necessitate matrix and precise spatial arrangement, demanding high‐throughput technologies to monitor differentiation. A research team observed ossification and vascularization after injecting “callus” organoids into nude mice for 4 weeks, suggesting their maturity and ability to attract vascular invasion. scRNA‐seq data compared with in vivo tissue indicated that the cells in the ‘callus’ organoids were in the late stage of osteogenesis within the cartilage, indicating their potential for promoting bone regeneration.^53^

### 1.8 Spatial transcriptomics sequencing

An ideal organoid model is supposed to be completely described with spatial information from the local environment of cells and its role in physiology. An Spatial profiling assays such as spatial transcriptomics technologies assess the tissue in situ organization and 3D architecture. This technology enhanced the quality control of organoid with multiplexed measurements, including identifying missing cell types and aberrant gene regulation.^7^, ^23^ Spatial transcriptome method reveals the mechanisms of multiple biological processes occur in different regions of the same tissue.

More convenient computer programs will be developed to assessing the spatial maps of organoids. Spatially Photo Activatable Color Encoded Cell Address Tags could preserve cell viability while annotating, tracking and isolating them. This method provides a computational framework to match the dataset with a large number of previous research data to determine the spatially biased transcriptome patterns and rich cell phenotypes.^54^

Coupling single‐cell methods with spatial analysis, cell atlas can generate cell signaling networks and transcription factor hierarchies. This method has been thoroughly tested in a long‐term organoid model and allows for complex cellular tests such as epithelium, mesoderm, and endothelial within 5−22 post‐conception weeks of pregnancy.^55^ (Figure 4D)

Taken together, single‐cell techniques offer unprecedented details to unveil biological processes at the molecular and cellular levels. In particular, scRNA‐seq detects heterogeneity, cell types and cell states in organoids, while scATAC‐seq recognizes and establishes adjustable sequences for these cell types. Spatial transcriptome approaches dissect spatial tissue atlases derived from transcript and protein abundances, providing comparisons to reference atlases to optimize organoid protocols. Advanced micro‐technologies, like microfluidics, can engineer organoids for high‐throughput functional analysis. Exhaustive assessment of cell intrinsic properties enhances our understanding of organoids as in vitro models for studying organ morphology and function.

### 1.9 Histological analysis

**FIGURE 5:**
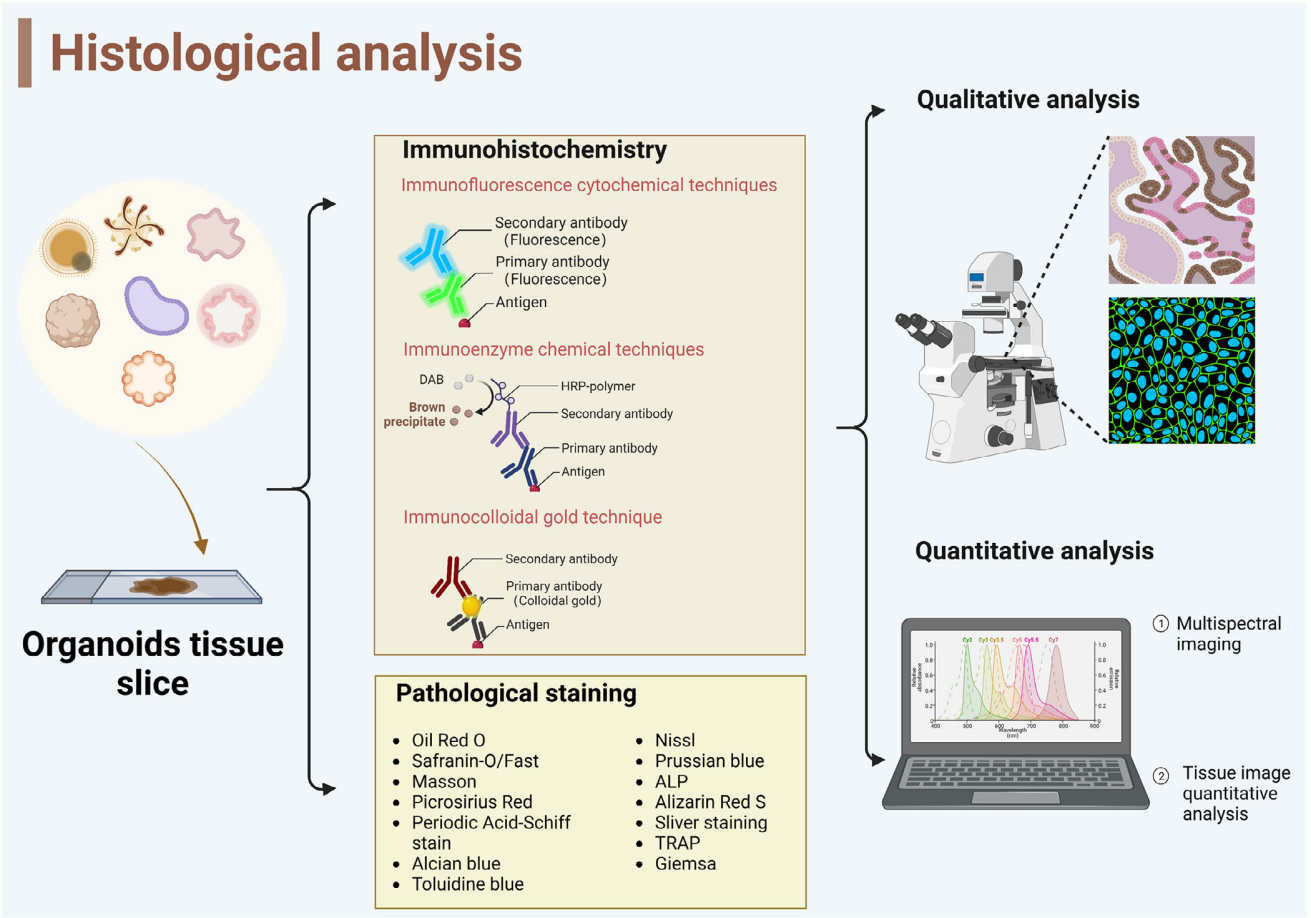
Histological analysis detects structural changes in organoids. Based on the antigen‐antibody binding reaction, histological analysis detects structural changes by pathological staining technologies. Through the special immunofluorescence combined with targeted cell types in organoids, the structure of the organoids could be seen clearly. (Created with BioRender.com).

Organoid simulates the similarity of tissue structures through the application of certain staining reagents that can be specifically combined with the chemical components of tissues/cells. The changes in the chemical composition are displayed by pathological analysis, thereby deepening the understanding of structural changes and metabolic changes, especially for the diagnosis of some metabolic diseases. (Figure 5) With physiologically relevant tumour models wildly used in recent studies, preclinical organoid models faithfully recapitulated the histological features and drug responses. Their importance in screening candidate drugs (especially chemotherapy drugs), as well as their applications in clinical progression, is gradually becoming apparent^56^ (Table 1). When using histological techniques to evaluate the growth of patient‐derived organoid (PDO), it was found that PDO has a spherical structure with an open lumen. It was also confirmed that PDO can express cholangiocarcinoma phenotype labeled cytokeratin by immunofluorescence technology, and its sensitivity to the drug NTRC 0652‐0 can be tested.^57^

**TABLE 1:** Representative histological analysis assessed the structure of organoids.

Histological analysis methods	Organoids	Origin	Structure	References
Hematoxylin/eosin (H&E) staining	Cerebral organoids	hiPSCs	Glioma patient‐derived cells implanted into brain organoids infiltrated growth	^56^
Immunofluorescence	CCA patient‐derived organoids	CCA patient‐derived	Grow as spherical structures with open lumens	^57^
Immunohistochemisty and in situ hybridization	GC organoid	Patient‐derived GC tissue	Modeling of histologic subtype specification in GC organoids	^59^
Immunostaining	Retina organoid	iPSCs	Formation of multilayered structure with pigment epithelial cells and outer segments	^60^
Immunostaining	Human liver organoid	hiPSCs	Unique micro‐anatomical architecture of HLO contained polarized human hepatocytes	^63^

Abbreviations: GC, gastric cancer; hiPSCs, Human induced pluripotent stem cells

Primary human organoids could display the morphology, tissue structure and polarity of tumour‐like cells, while the cell spheroids can only form a homogenous, uniform non‐hollow sphere.^58^ Histologic subtype specification in patient‐derived gastric cancer (GC) organoids reflects a clonal difference or differentiation hierarchy. Characterized by in situ hybridization of histopathologic analysis and gene expression analysis, human diffuse GC was established to accurately model the histologic subtypes. This suggested that the histologic subtype specification was regulated by tumour microenvironment rather than cell‐intrinsic differences regulates.^59^

Human organoids also recapitulate the diversity and function of target organ, which could evaluate by histological analysis for basic and translational research. scRNA‐seq and histochemistry reveal retina organoids’ similarity to human retina cell types and in vivo development.^60^ They contained patches of pigment epithelium and their photoreceptors displayed characteristic subcellular compartments, which had matched formation of the adult retina with three nuclear layers and two synaptic layers. Bioreactors can scale up the manufacture of retinal cells based on these features.^61^

Morphological or histochemical analysis can detect cell hierarchy when continuously culturing for long time to observe differentiate direction.^62^ Histological and ultrastructural analyses are valuable for evaluating the microanatomy structure of the human liver organoid (HLO) drug screening model. Immunohistochemical analysis revealed tubular structures in HLO containing human liver cells, mirroring the unique micro‐anatomical structure of hepatic tissue in vivo. To advance investigations, HLO‐based drug‐induced liver injury detection can be transformed into a high‐speed real‐time imaging platform with 384 wells, enabling multi‐channel reading for measuring survival ability.^70^ This approach successfully predicted genomic predisposition for liver toxicology studies and facilitated compound optimization.^63^

Histological assessment of organoids has inherent limitations, especially when evaluating pathway‐specific neuronal network activities in brain organoids. Alternative approaches like calcium imaging and extracellular recording are needed to overcome these limitations and gain deeper insights into brain network functions.^64^ Poor tissue integrity affects evaluation in tendon organoids, necessitating research into rejuvenation strategies.^65^

### 1.10 Morphological analysis

**FIGURE 6:**
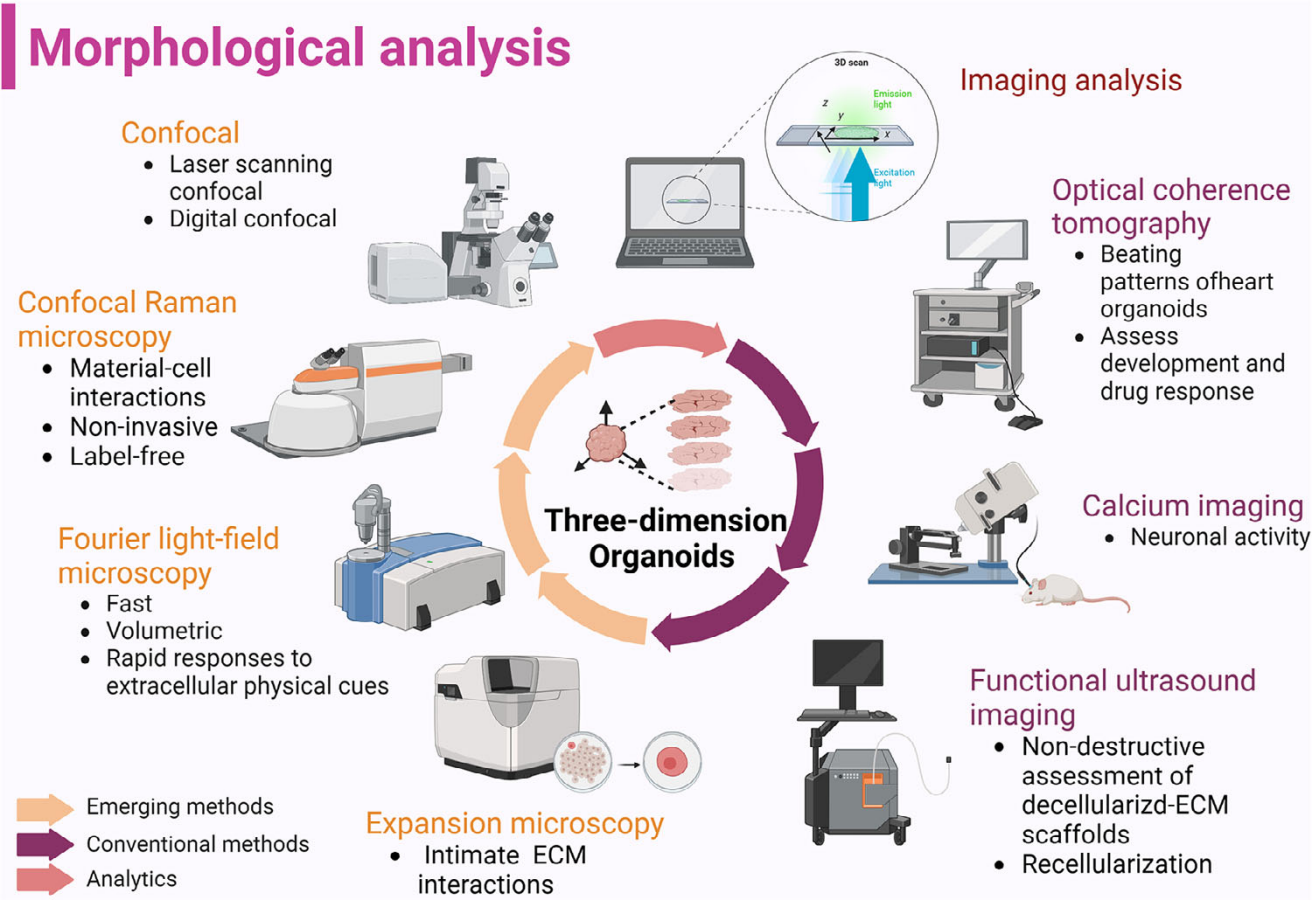
Recent morphological analysis used in imaging of organoids. To decode the 3D structure of organoids, not only conventional technologies are applied in the characterization, but also high‐content imaging systems were used to fully display the comprehensive special information. (Created with BioRender.com).

Technical advances in microscopy make it possible to screen and image increasingly complex biological models . To this end, organoids are expected to be used for high‐content imaging because recapitulating their mother tissue requires well‐characterized. Meanwhile, image‐based selection based on microscopes provides high information content and allows for phenotype analysis. Nonetheless, obtaining high‐resolution images and analyzing complex organoid structures pose technical and analytical challenges^66^ (Figure 6).

To visualize spatial relationships of organoids, fluorescence live microscopy accompanied with scanning electron microscopy can be used to assess.^67^ When assessing 3D morphologies and cellular densities of organoid, optical coherence tomography (OCT) is a useful method to detect the influence of Matrigel on organoid generated via liquid overlay technique without labeled. Furthermore, OCT not only provides information of construct viability but also nominates a proof‐of‐concept for longitudinal drug efficacy studies by discerning live or dead cells within both disk‐like aggregates and tumour organoids^68^ (Table 2).

**TABLE 2:** Representative promising imaging methods applied in organoid.

Morphological analysis methods	Organoids	Origin	Function	References
Optical coherence tomography	Multicellular tumour spheroids	Human breast cancer cells	Longitudinal visualization and tumour aggregate with/without 3D spheroids in Matrigel	^68^
Fluorescence lifetime imaging microscopy; hyperspectral imaging	Retinal organoids	Human stem‐cell	Examine the microstructure and metabolic function of living organoids	^70^
Phototransfer by allyl sulfide exchange‐expansion microscopy (PhASE‐ExM)	Intestinal organoids	Murine small intestinal crypts	Optical clearance and super‐resolution imaging of organoids and their ECM	^71^
Hybrid point spread function (hPSF‐FLFM)	hiPSC‐derived colon organoids	hiPSC	Enhanced optical sectioning and contrast	^72^
3D functional ultrasound imaging	Human liver organoids	Decellularizing of Wistar rats’ livers matrix scaffolds	Assessing vascular network structures and nutrient acquisition in organoids	^78^
Inducible cell division counter (iCOUNT)	Forebrain organoid	Neural stem/progenitor cells	Live imaging and analyze the cell division history	^79^
High‐speed scanning ion conductance microscope	Metastatic intestinal organoids	Mouse intestinal tumour	Long‐term imaging and mapping nanomechanical properties of basal surfaces	^80^

Abbreviation: ECM, extracellular matrix.

Besides visualization of organoid growth, confocal Raman microscopy was used to evaluate organoid formation within the gel matrices in recent study. This label‐free and non‐invasive technique provides valuable information about material‐cell interactions, facilitating the selection of suitable hydrogel formulations as animal‐derived cultivation matrix substitutes.^69^ Furthermore, phase contrast microscopy, fluorescence lifetime imaging microscopy (FLIM), and hyperspectral imaging (HSpec) provide the opportunity for real‐time and non‐invasive analysis of the microstructure and metabolic functionality of living organoids.^70^ Limited by penetration depth of depth‐dependent light attenuation, organoids are difficult to characterize the intimate cell‐ECM interactions. Thereby, phototransfer by allyl sulfide exchange‐expansion microscopy (PhASE‐ExM) is developed to overcome these challenges even in sub‐micrometer scale to visualizing organoids and their ECM.^71^ Traditional imaging methods inevitably affect the temporal resolution of volumetric acquisition, resulting in the inability to capture rapid cellular and tissue dynamic changes. The hybrid point spread function (hPSF‐FLFM) and Fourier light field microscope can be used to scan more quickly with larger volume and high resolution and explore the complex spatiotemporally mechanism in organoid research.^72^ FLIM was also used to visualize living mechanical stress when organoids‐on‐a‐chip subjected to flow.^73^, ^74^

Various imaging methods have transformed organoid assessment. Confocal microscopy facilitates drug penetration and real‐time fluorescence quantification for drug response analysis in retinoblastoma vitreous seeds.^75^ Live imaging dyes, a tool of live functional assays can reveal longitudinal maturation of transepithelial transport in kidney organoids.^76^, ^77^ Gessner et al. introduced a 3D functional ultrasound imaging technique that provides insights into vascular network structures and nutrient acquisition in organoids, particularly valuable for human liver development. The method offers statistical flow velocity distribution visualization, showing the matrix of vascular circuits, and has potential for hemodialysis research using ‘biological matrix scaffolds’ with preserved collagens and growth factors.^78^

Chronic imaging is not easily compatible with methods for molecular phenotype screening by isolating cells. Denoth‐Lippuner et al. invented an inducible cell division counter (iCOUNT) for analyzing the cell division history of neural stem/progenitor cells in vitro using delayed imaging and recombinant protein induced label exchange with cell‐cycle‐dependent endogenous proteins labeling.^79^ High‐speed scanning ion conductance microscope for long‐term imaging is utilized to simultaneously reveal morphologies of organoids. It can dynamically reveal the morphological correlation factor depending on cell types between basal surface of cells and local elastic modulus (E).^80^

Long‐term time‐lapse imaging enables monitoring the viability and homogeneity of multiple organoids in parallel.^81^ Analyzing the structural development of organoids is challenging due to their heterogeneity and abstract morphology. Abdul et al. introduced D‐CryptO, a deep learning‐based image analysis tool, to automatically classify organoid morphology, particularly evaluating crypt formation and opacity of colorectal organoids, addressing this limitation effectively.^82^

Cerebral organoids (or brain organoids) offer a valuable platform for live assays, including fluorescence imaging. Giandomenico et al. devised a simple yet effective approach, combining basic tools and immune staining, to label neurons and study their morphology. This scheme highlights the structural details of organoids, resulting in improved morphology and reproducibility compared to unpatterned brain organoids. Due to its high signal‐to‐noise ratio, this method is very suitable for high contrast segmentation research.^6^

The lack of a vascular system in brain organoids leads to the formation of a necrotic core, limiting their usefulness as a model for drug screening and spatial patterning experiments. Addressing this problem, an engineering approach using a polycaprolactone scaffold creates flat organoids with improved diffusion conditions, preventing necrosis. This method allows customization of size and facilitates self‐generated gyrification for better brain modeling.^83^ Another study used a millifluidic system with continuous media flow, reducing ‘dead cores’ and enhancing cell vitality and differentiation efficiency through improved oxygenation and metabolic maturation.^84^

These advanced imaging technologies offer insights into organoids. By bridging the gap between cellular dynamics and macroscopic behavior, these imaging tools pave the way for advancements in organoid‐based studies, drug screening and tissue engineering. However, morphological analysis limited in dynamic data, potential artifacts and subjectivity. More advanced methods of image processing and interpretation have yet to be developed.

### 1.11 Functional properties analysis

**FIGURE 7:**
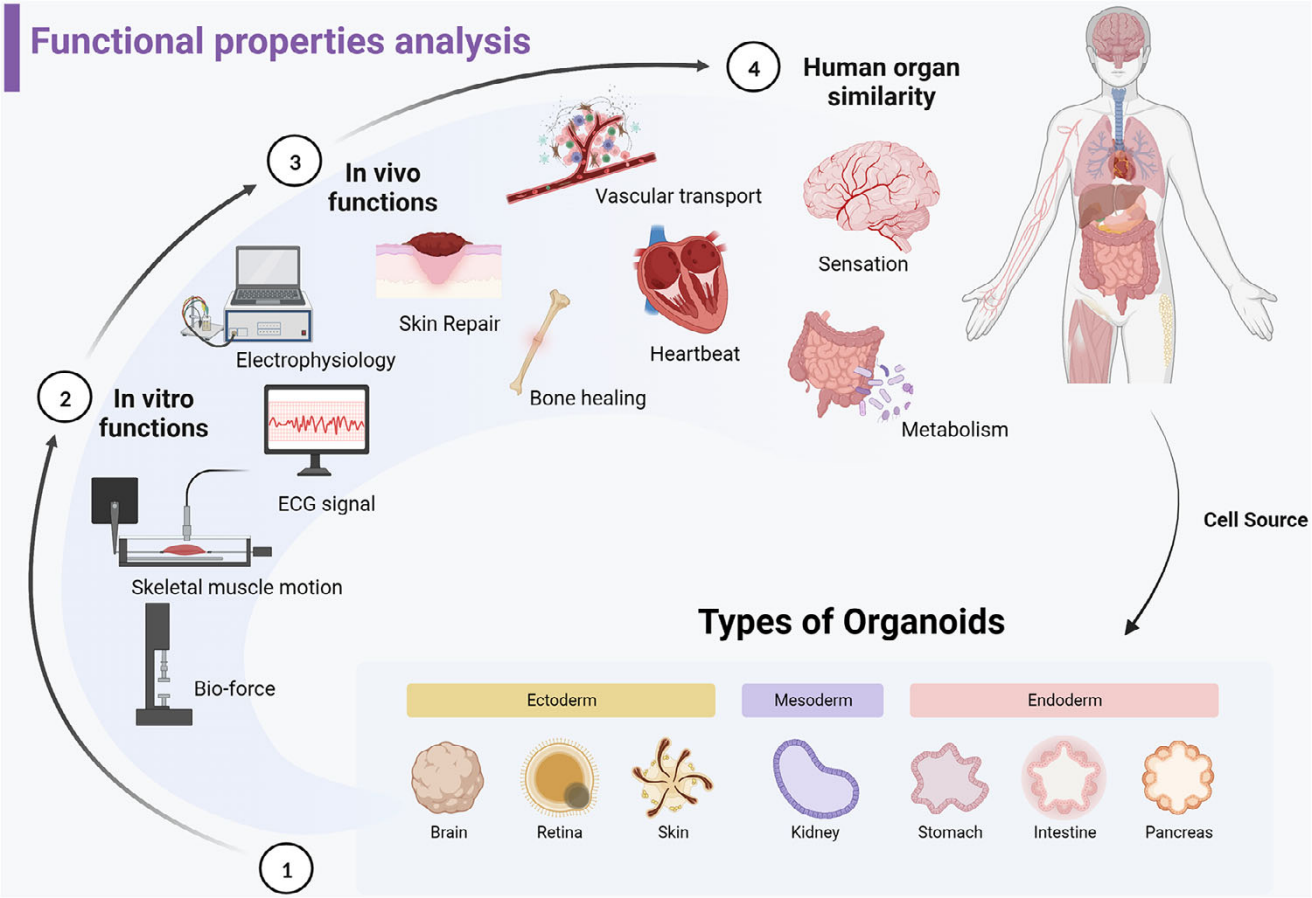
Functional properties analysis evaluates the simulacrum of organoids. Different types of organoids were generated from stem cells, with basial functional properties both in vitro and in vivo. More and more technologies combined with conventional devices were developed to assess the physiology and structural functional properties, such as electrophysiology and biomechanics in vitro. Importantly, organoids should be developed to mimic microcosmic biological process and macroscopic systematic process in vivo in the future studies. (Created with BioRender.com).

Considering the multiple functions of human real tissues and organs, organoids are supposed to have similar functions (Figure 7). While fully simulating functional properties remains challenging, specific analyses can evaluate their similarity. A living biobank of well‐characterized bladder cancer organoids from 53 patients revealed diverse subtypes through immunohistochemistry and gene expression analysis, suggesting potential for developing new bladder cancer treatments.^85^ This highlights the future direction of organoid development, aiming to create a multitude of in vitro models with distinct functional properties, resembling biobanks. These advancements in organoid technology open doors for personalized medicine, drug discovery and disease modeling.

#### 1.11.1 In vitro

To better fit the real organ of human body, simulating the corresponding physical properties in vitro is a further requirement for building mature organoids.

##### Physiology functional property

CMOS‐based microelectrode array (MEA) devices enable large‐scale mapping of cortical synaptic projections and functional connections among neural networks. High‐density 3D electron probes overcome technical limitations, recording three‐dimensional neural activity. Advanced CMOS‐MEA technology generates detailed electrical activity maps from organoid slices, equivalent to forming brain cross‐sectional areas. Sharf et al. allocate individual unit activity precisely, showing pharmacological perturbations' impact on physiological parameters using electrode redundancy and waveform shapes determined by neuronal location. Innovations in neural activity recording offer valuable insights into organoid functionality, enhancing our understanding of neural networks and drug effects.^86^

**FIGURE 8:**
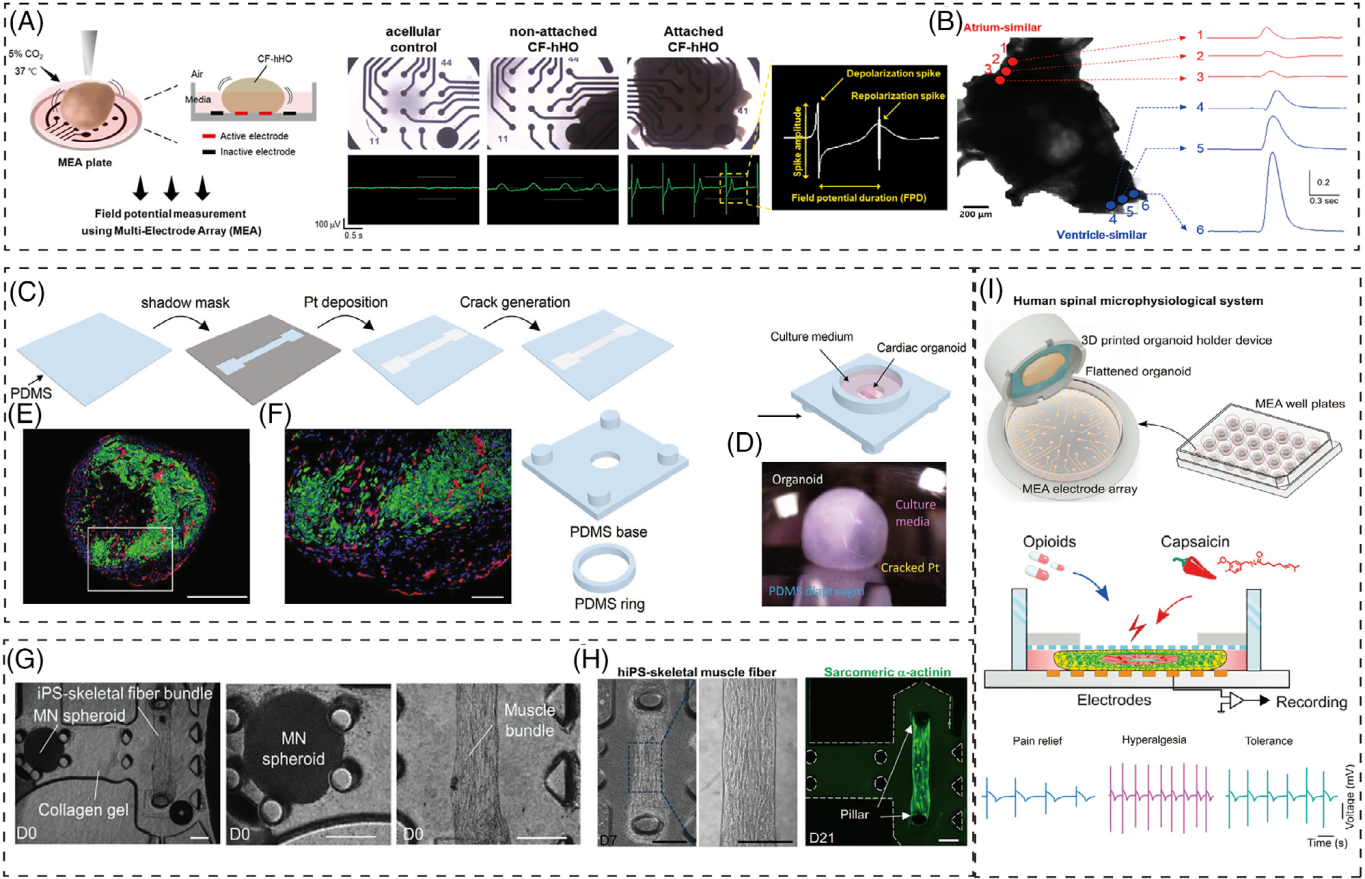
Advanced technologies evaluate the multiple functional properties of organoids. (A) Verification of maturation of cardiomyocytes (CMs) in beating chamber formation‐ human heart (CF‐hHOs).^87^ Copyright 2022, Elsevier. (B) Local waveforms of intrinsic optical sensing (IOS) in atrium‐similar area (red) and ventricle‐similar area (blue).^87^ Scale bar: 200 μm. Copyright 2022, Elsevier. (C) Pt‐based elastic nanocracked force‐sensing diaphragm assembled in a polydimethylsiloxane (PDMS) ‐based organoid culture chamber.^89^ Copyright 2022, Springer Nature. (D) Optical image of organoid and the soft force sensing diaphragm interface.^89^ Copyright 2022, Springer Nature. (E and F) Engineered vascularized human cardiac organoid stained with cardiac troponin T (green, cardiomyocytes), CD31 (red, endothelial cells) and DAPI (blue, nuclei).^89^ Scale bars in (E) and (F) are 500 and 100 μm. Copyright 2022, Springer Nature. (G) Microphysiological 3D model of amyotrophic lateral sclerosis (ALS) and optogenetic motor neurons. Motor neuron (MN) spheroid and a muscle fiber bundle were embedded in collagen gel of a microfluidic chip on day 0 (D0).^91^ Copyright 2018, American Association for the Advancement of Science. (H) Fabricated skeletal muscle fiber bundle approximately 1500 μm in length attaching the pillars at D7 and D21.^91^ Scale bars, 200 μm. Copyright 2018, American Association for the Advancement of Science. (I) Human spinal microphysiological system consisting of human spinal cord organoid and 3D printed organoid holder device to model opioid‐induced tolerance and hyperalgesia.^93^ Copyright 2022, Elsevier.

According to recent researches, it can be seen that heart organs have gradually developed the basic morphology of chamber and cardial cell tissues and have the function of simulating vascularization and electrophysiological signals^87^ (Figure 8A‐B). Compared with patch clamp which analyzes action potential of single cells with micro tip, less damage to organoids measured with calcium imaging and MEA of electrophysiology signal. Consequently, the advent of new technologies providing high‐throughput and multimodal characterizations may be wildly used in organoids in the future.^88^

##### Structure functional property

Optical imaging is limited for mechanical assessment of heart tissue or organoids. Integrating soft electronic sensors based on nano split platinum film provides a soft and ultrasensitive method to directly measure heart organoid contractions in natural cell culture. This technology allows for reliable contact with organoids of various sizes and shapes, offering valuable insights into their mechanical properties and function^89^ (Figure 8C‐F).

The contractility of skeletal muscle tissue can be evaluated by image‐based methods to characterize the movement of soft structures. In order to accurately measure the spatial and temporal contraction mechanical properties related to optical stimuli, Zhao et al. created a type of micro 3D framework. As a mechanical interface for 3D skeletal muscle tissue, the 3D photo genetic active muscle ring can detect the mechanical properties of skeletal muscle tissue under various conditions.^90^ Motor neuron spheroids generated from iPSCs cocultured with skeletal muscle fibers to induce muscle contraction in a microfluidic device to determine the pathogenesis of amyotrophic lateral sclerosis^91^ (Figure 8G‐H). Other mechanical properties of multiple organoids are waiting for further functional assessing methods to characterize.

#### 1.11.2 In vivo

The ability of organoids to mimic biochemical processes in vivo is not yet robust and requires additional techniques to assess and detect.

##### Microcosmic biological process

Brain organoids represent a promising approach for patient‐matched brain repair. In order to study the anatomical performance of brain neural circuits, optogenetics has been used to study organoid nerves.^92^ Yoon et al. applied optogenetics stimulation to human cortical spheroids (hCS) expressing ChR2 to trigger potential effects. The results show that hCS is functionally mature and can simulate the pathogenesis of nervous system disease.^93^

Recent advances in modeling human pain are promising; however, reproducible neural electrical measurements of spinal cord organoids are still difficult to achieve. The new human spinal cord microphysical systems, combined with plug‐and‐play neural activity sensors, have achieved modeling of the biological processes of opioid induced tolerance and allergic pain. The unique flat organoid design not only overcomes the electrode contact problem between organoids and MEA electrodes but also supports neuronal maturation/activity, which would be broad applied in translational pain research^94^ (Figure 8I). Additionally, Wu et al. developed a taste organoids‐on‐a‐chip system (TOS) with bioelectronic organoids to simulate the taste system. The TOS accurately recognizes taste sensations by extracting signal features from stimuli concentrations and using principal component analysis.^95^ These innovations hold potential for advancing pain and taste research.

##### Macroscopic systematic process

Current gut‐like organoids still lack full representation of human intestinal biology and immune components in vivo. A new human intestinal organoid with immune cells has shown activation of immune response and IgA antibody secretion upon microbial exposure, providing a model for studying infectious or allergen‐driven intestinal diseases.^96^

The direct integration of liver organoids with liquid chromatography‐MS (LC‐MS) allows for selective automatic tracking of drug metabolism. The liver organoids are equipped with liquid chromatography column housings, which can monitor and analyze drug phase 1 metabolism by coupling the ‘organ‐in‐a‐column’ unit with LC‐MS online. At the same time, as proof of concept, the ‘organ‐in‐a‐column’ unit can detect the production of enzymatic metabolism of heroin.^97^

Maturation of organoids is a major challenge. Although organoids are able to differentiate into a diversity of many cell types, the capacity to reveal physiologically related functional, given potentially imprecise specification and maturation state, is still unknown.^98^ However, with more advanced technologies, organoid will be the ideal model mimicking both in vitro and in vivo functional properties.

## 2 CONCLUDING REMARKS

Over the decades, organoids have undergone a transition of technologies limitations in constructing organoids to further assessing the physiological fidelity to justify their utility. Researchers have made substantial efforts to improve the fidelity of organoids by optimizing refinements of the culture format and conditions, such as organ‐on‐a‐chip applied in high throughput drug screening.^99^ However, as diverse protocols seem to generate organoids with inconsistent features, it is still urgent to put forward gold standards to define what ‘true’ organoids are.

The technologies discussed in this paper offer high‐information content to explore the cellular mechanisms within multicellular structures of organoids, enabling assessment of their composition and functional properties compared to human tissues in vivo. Traditional low‐dimensional techniques elucidate the genomics, proteomics and epigenomics of organoids, while advanced single‐cell methods allow for assessment of subtle cell states and spatial information.^24^ Pathological and morphological analyses provide macroscopic insights, essential for understanding the physiological relevance of organoids. Characterizing cellular composition, histoarchitecture, morphological structure and functional characteristics ensures a reproducible and comprehensive organoid model.^100^ However, considering tissue heterogeneity, can organoids fully replace conventional animal models even in functional properties? Future assessments must enhance fidelity in both molecular mechanisms and unique physical characteristics. On the other hand, we have to keep in mind that a robust organoid model is essential to yield reproducible results rather than 3D cell mixture system for basial and translational studies. A robust organoid model should mirror cellular organization, tissue structures, gene expression patterns and developmental trajectories of in vivo human organs.

**FIGURE 9:**
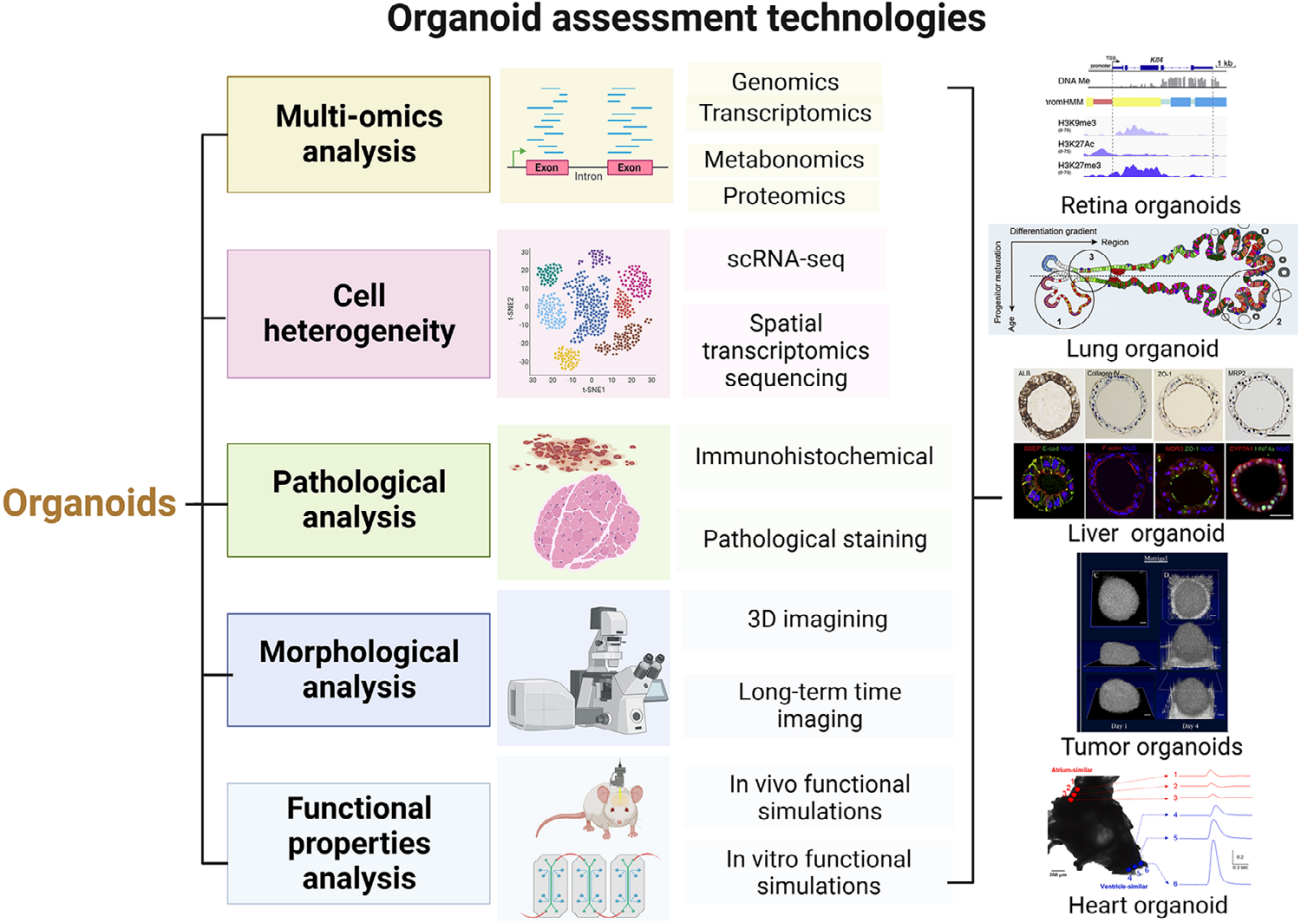
The scheme of technologies for comprehensive assessments of organoids. A future catalog of well‐assessed organoids will focus on quantifying biological variation and addressing environmental perturbations to better represent primary tissue in vivo. To fully assess the complex organoid models, multi‐omics analysis plays a key role in revealing molecular mechanisms within organoids, analyzing the genome, transcriptome, and proteome. Single‐cell analysis and spatial profiles yield an unprecedented high‐dimensional assessment of molecular maps within organoids compared to corresponding human tissues. Pathological and morphological analysis restore the full picture of architectures on a macro‐scale to assess the similarity. Functional properties analysis will determine if organoid protocols achieve complex functions resembling real organs.

In summary, with the continuous expansion of the application scope of multiplexing multimodal technology, organoids are gradually becoming an accurate and universal biomimetic model. With the widespread integration of intelligent algorithms and mechanism research in organoids,^23^ high‐dimensional technologies will be developed to improve the biomimetic accuracy of organoids. Perhaps organoid co‐cultures will also be used for larger scale cell‐cell communication and systematic biological process modeling to generate biological insights in the near future (Figure 9).

## AUTHOR CONTRIBUTIONS

Yuyuan Gu, Wencai Zhang and Xianmin Wu conceived, wrote, revised the manuscript and made the figures. Yuanwei Zhang revised the manuscript and figures in the process of revision. Ke Xu and Jiacan Su reviewed and edited the manuscript. All authors read and approved the final manuscript.

## CONFLICT OF INTEREST STATEMENT

The authors declare no competing interests.

## ETHICS APPROVAL AND CONSENT TO PARTICIPATE

Not applicable.

## Data Availability

Data availability is not applicable to this article as no new data were created or analyzed in this study.
